# Emerging insights into the relationship between hyperlipidemia and the risk of diabetic retinopathy

**DOI:** 10.1186/s12944-020-01415-3

**Published:** 2020-11-19

**Authors:** Yuyu Chou, Jin Ma, Xin Su, Yong Zhong

**Affiliations:** 1 Department, of Ophthalmology, Peking Union Medical College Hospital, Chinese Academy of Medical Sciences & Peking Union Medical College, Beijing, 100730 China; 2 Department of Cardiology, The Xiamen Cardiovascular Hospital of Xiamen University, Xiamen, 363001 Fujian China

**Keywords:** Hyperlipidemia, Diabetic retinopathy, Lipid profiles, Lipid-lowering treatment, Pathophysiology, Statin, Fibrate, Inflammation

## Abstract

Hyperlipidemia is correlated with a series of health problems. Notably, aside from its established role in promoting cardiovascular morbidity and mortality, hyperlipidemia has also been considered for modulating the risk and the severity of multiple metabolic disorders. According to the results of epidemiologic investigations, several certain circulating lipoprotein species are correlated with the prevalence of diabetic retinopathy, suggesting that the physiological and pathological role of these lipoproteins is analogous to that observed in cardiovascular diseases. Furthermore, the lipid-lowering treatments, particularly using statin and fibrate, have been demonstrated to ameliorate diabetic retinopathy. Thereby, current focus is shifting towards implementing the protective strategies of diabetic retinopathy and elucidating the potential underlying mechanisms. However, it is worth noting that the relationship between major serum cholesterol species and the development of diabetic retinopathy, published by other studies, was inconsistent and overall modest, revealing the relationship is still not clarified. In this review, the current understanding of hyperlipidemia in pathogenesis of diabetic retinopathy was summarized and the novel insights into the potential mechanisms whereby hyperlipidemia modulates diabetic retinopathy were put forward.

## Introduction

Hyperlipidemia, characterized by reduced circulating high-density lipoprotein cholesterol (HDL-C), elevated circulating low-density lipoprotein cholesterol (LDL-C), and very low-density lipoprotein cholesterol (VLDL-C), has been verified to be closely correlated with a series of cardio-metabolic disorders, such as obesity, hypertension, and coronary heart disease [1]. For instance, the process whereby excessive LDL-C intrudes into the sub-endothelium is intimately involved in the cascade to atherosclerotic lesions; meanwhile, reduced circulating LDL-C levels retard the process of atherosclerosis [2]. On the other hand, the HDL particle displays a vital anti-atherosclerotic effect since it accepts cholesterol from peripheral tissues for delivery to the liver through a classical method that is called reverse cholesterol transport (RCT). Additionally, the well-established anti-oxidative function of HDL, which prevents the oxidation progression of LDL particle into ox-LDL particle, also inhibits the initial atheroma formation within the sub-endothelial region [3]. Thereby, hyperlipidemia has become a pressing issue which poses serious risks to health of human beings.

Emerging evidence shows that aside from its established relationship with cardiovascular diseases, hyperlipidemia has been confirmed for the correlation with diabetes mellitus and its various complications, such as diabetic retinopathy [4]. Recently, hyperlipidemia is considered as one of the strongest risk factors for pathogenic development of diabetic retinopathy [5]. As reported, diabetic retinopathy is one of the most significant complications in diabetic patients and meanwhile, it is also considered as one of the essential causes of visual damage, hand in hand with the sight-threatening disease in around 20% of diabetic patients, leading to more than 45,000 novel cases of blindness among American per year [6]. In the clinical practice, diabetic retinopathy has two major types, as non-proliferative diabetic retinopathy (NPDR) and proliferative diabetic retinopathy (PDR). To be more specific, NPDR, identified as the earliest stage of diabetic retinopathy, is characterized by having severe retinal vascular permeability, micro-aneurysms formation, exudation, hard exudate, and hemorrhage. Under this condition, atrophy and ischemia of the neurosensory retina resultantly induce severe vision loss. The further advanced stage of PDR is characterized by nerve-fiber layer infracts, neo-vascular proliferation, vitreous tortuosity and hemorrhage, resulting in increased risk of visual impairment due to tractional retinal detachment and hemorrhage [7].

Two of the classical risk factors of diabetic retinopathy, including hyperglycemia and hypertension, are strongly involved in the pathogenic development of diabetic retinopathy [8]. The clinical managements of those two aberrant conditions have been demonstrated in randomized trials which significantly prevent the conception of diabetic retinopathy without diagnosed, as well as reduce the development of diabetic retinopathy and the visual loss when it is already existed [9]. Thus, several international clinical guidelines focusing on diabetic retinopathy therapy have paid attention to the strict control of hyperglycemia and hypertension [10, 11]. Nonetheless, being more common than in the general population after correction for the traditional risk factors, the increased incidence of diabetic retinopathy may potentially be explained by other risk factors. Importantly, the relationship between hyperlipidemia and diabetic retinopathy has been under investigation, but a conclusive linkage is still not elucidated. In the past decades, the lipid-lowering agents have been identified as the potential therapeutic agents for diabetic retinopathy, and the management of hyperlipidemia could be an additional aim in patients with diabetes [12]. Herein, in the current paper, the understandings of hyperlipidemia in the pathogenesis of diabetic retinopathy have been put forward. Moreover, the novel insights into the potential mechanisms by which hyperlipidemia regulates the risk of diabetic retinopathy have also been summarized.

## Classification and clinical significance of serum lipid profiles

Serum lipid profiles are classically classified into two main types, as the simple lipids and the complex lipids. Simple lipids contain total fatty acid and cholesterol, while complex lipids contain total triglyceride (TG) and cholesterol ester [13]. In humans, TG are always packed after feeding both in the small intestine as chylomicrons (CM) and in the liver as VLDL [14]. With in-depth investigations, these lipoprotein complexes contain a hydrophobic essential core of the cholesterol ester and the TG ester, which is surrounded by a hydrophilic configuration of proteins and phospholipids for solubility [15].

The serum lipoprotein complexes are known collectively as apolipoproteins, including apolipoprotein A1 (ApoA1), apolipoprotein C3 (ApoC3), apolipoprotein B (ApoB), and apolipoprotein A5 (ApoA5) [16]. Notably, the lipoproteins are classified according to their density, the content of lipids, and the composition of diverse apolipoproteins. The LDL-C carries up approximately 70% to 75% of total cholesterol [17].

Recently, evidence has illuminated that the role of circulating lipid profiles on affecting the risk of cardio-metabolic disorders. As reported, the circulating level of LDL-C is the major contributor to the promotion of atherosclerosis and is the strongest predictor of atherogenesis [18]. Thereby, it has been firmly established for the relationship between circulating levels of LDL-C and risk of atherosclerosis; meanwhile, it is not doubt about the benefits obtained from the lipid-lowering treatments. Till now, several large-scale randomized controlled trials from different countries have shed light on the confirmed beneficial effects of lipid-lowering agents, such as statin, ezetimibe, and fenofibrate, in reducing the risk of atherosclerotic related cardiovascular disease, making the lipid-lowering treatment become the most important of therapeutic strategy in reducing the prevalence and the severity of major adverse cardiovascular events (MACE) [19]. Consistent with this notion, several important guidelines have flagged LDL-C as an important therapeutic target for patient with diabetes.

It has also been recommended that the serum apolipoprotein B-100 (ApoB-100), as the only apolipoprotein within LDL particle, could be used to predict the cardiovascular risk [20]. On the contrary, multiple clinical investigations have confirmed that higher serum levels of HDL-C are closely correlated with a lower risk of MACE. According to these eye-catching results, it has been proposed to use the inhibitors of cholesteryl ester transfer protein (CETP) in order to up-regulate the circulating HDL-C levels, such as using Torcetrapib, Evacetrapib, and Dalcetrapib. However, the results showed that the treatment with inhibitors of CETP could not obtain expected benefits [21].

## Clinical trials on hyperlipidemia and diabetic retinopathy

Several clinical trials containing diverse sample of participants focus on the correlations between hyperlipidemia and diabetic retinopathy. However, the published results are inconsistent. The discordances in results might be ascribed to the various measurements of detection for patients with diabetic retinopathy and the diverse standards of diagnosed definitions for diabetic retinopathy.

As described above, the potential role of hyperlipidemia in regulating diabetic retinopathy has begun to gain appreciation, whereas the majority of published papers have demonstrated no statistical significant relationship between serum lipid profiles with the development of diabetic retinopathy. For instance, the Wisconsin Epidemiologic Study of Diabetic Retinopathy (WESDR), which enrolled 2,366 diabetic subjects, assessed the risk biomarkers for diabetic retinopathy and revealed that the serum total cholesterol had no significant correlation with the development of diabetic retinopathy [22]. Additionally, the serum total cholesterol has also been confirmed as a biomarkers for severity of hard exudate in the individuals with young-onset diabetes mellitus. By contrary, within the participants with older-onset diabetes, the circulating levels of total cholesterol were not related with the severity of diabetic retinopathy and hard exudate [23].

Likewise, using the same cohort, the authors conducted another investigation which went on a subsequent 5-year follow-up and found a significant relationship between the incidence of retinal lesions and serum levels of LDL-C, whereas multivariate correlations focusing on the additional risk factors did not show any association [24]. It is worth noting that the WESDR trial did not assess the serum levels of TG or differentiate between LDL-C and HDL-C levels, thereby it could not conclude whether hyperlipidemia promote the severity of diabetic retinopathy. The similar results could be observed in another independent study, named the Sorbinil Retinopathy Trial (SRT), which also failed to discover the relationship between hyperlipidemia with the pathological promotion of diabetic retinopathy in the subjects with type 1 diabetes mellitus [25, 26].

Interestingly, the relationship was further investigated in many studies. However, the results did not reveal an accurate relationship. For instance, a study that focused on the Tehran subjects, evaluated the risk factors of diabetic retinopathy. By analysis, the authors found that among 8,000 enrolled subjects, about 10% of them presented diabetes, but the prevalence of hyperlipidemia had no obvious correlation with the risk of diabetic retinopathy [27, 28]. Consistently, another large-scale clinical trial, called the Australian Diabetes, Obesity and Lifestyle Study (AusDiab Study), screened about 2,200 participants with diabetes mellitus and found that the circulating levels of LDL-C were not correlated with the development of diabetic retinopathy [29]. In contrast, a study named the Singapore Malay Eye Study (SMES) put forward that the higher body mass index (BMI) and higher circulating levels of LDL-C were related with the pathogenic development of diabetic retinopathy; notably, these results were subsequently replicated in another independent study, indicating an associations between BMI and diabetic retinopathy [30].

On the other hand, increasing evidence explains hyperlipidemia with the presence of the complications of diabetic retinopathy, such as hard exudate and diabetic macular edema. According to the previous conclusions, the hard exudate, an early symbol of diabetic retinopathy, has been considered to be induced by lipid leakage from dysfunctional retinal capillaries. In the WESDR trial, the serum levels of total cholesterol were correlated with the risk and the severity of hard exudate among the participants with young-onset type 2 diabetes mellitus [22, 23]. In the Early Treatment of Diabetic Retinopathy Study (ETDRS), serum lipid profiles were also confirmed to be correlated with hard exudate. In details, the elevated serum levels of total cholesterol induced about two-fold elevated risk of diabetic retinal hard exudate in the subjects with both type 1 and type 2 diabetes mellitus, and the subjects with higher circulating LDL-C were also more inclined to suffer from diabetic retinal hard exudate. Synchronously, the higher circulating levels of total cholesterol, LDL-C, and TG could be considered as biomarkers for the development of diabetic retinal hard exudate [31]. In another independent population-based trial, named the Chennai Urban Rural Study (CURES), the subjects with diabetic retinopathy presented increased circulating levels of TG compared with the healthy individuals, and the circulating levels ofLDL-C was significantly correlated with the risk of diabetic macular edema [32]. In another large-scale clinical investigation, the author found that the risk of hard exudate could be induced and deteriorated by higher serum LDL-C levels [33]. More recently, The Sankara Nethralaya Diabetic Retinopathy Epidemiology and Molecular Genetic Study (SN-DREAMS) also demonstrated that higher circulating LDL-C levels and higher total cholesterol/LDL-C ratio were correlated with diabetic macular edema [34].

**Fig. 1:**
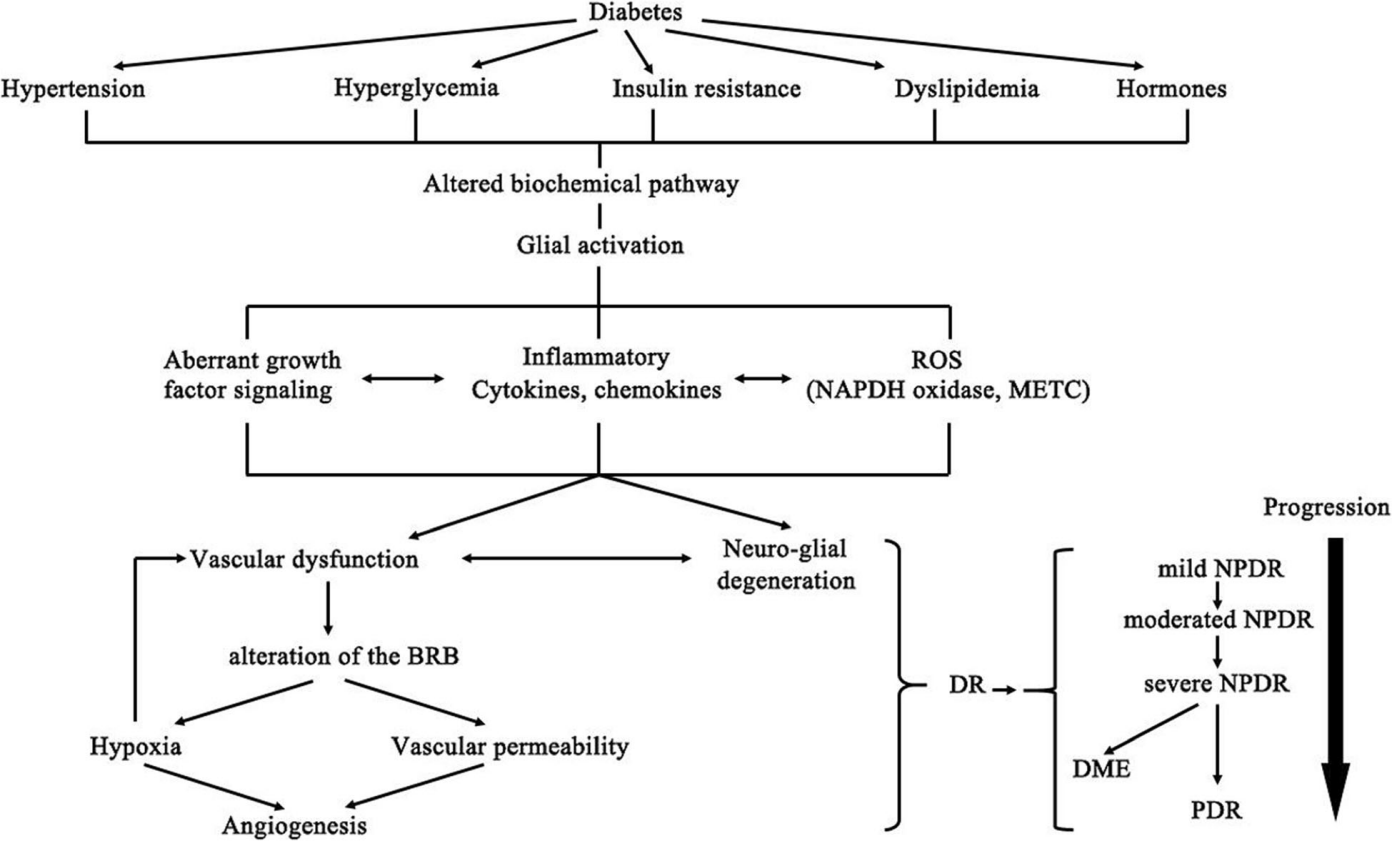
Figure of the potential mechanisms which induce sight-threatening events of diabetic retinopathy (DR), proliferative DR (PDR), and diabetic macular edema (DME). Abbreviation: AGE, advanced glycation end-products; PKC, protein kinase C; RAS, renin– angiotensin system, ROS, reactive oxygen species; NADPH, Nicotinamide adenine dinucleotide phosphate; mETC, mitochondrial electron transport chain

Clinical trials on lipid markers and diabetic retinopathy have been summarized in Table 1. Given that the published results are discordant, more trials with large-scale sample of participants are still needed to further explore the relationship between hyperlipidemia and diabetic retinopathy Fig. 1.

**Table 1:** Clinical trials on lipid markers and diabetic retinopathy

Study, year, references	Reference	Participants Size	NOS score	Quality	Major Results	Biomarkers of serum lipid profiles			
						TC (mmol/L)	TG (mmol/L)	LDL-C (mmol/L)	HDL-C (mmol/L)
	Cross-sectional studies								
EURODIAB, 2008	[35]	2,991	8	High	NPDR	5.42 ± 1.11	1.22 ± 0.82	3.25 ± 1.10	1.76 ± 0.45
					PDR	5.51 ± 1.51	1.36 ± 1.08	3.12 ± 0.94	1.65 ± 0.51
WESDR, 2008	[22]	2,366	8	High	Hard exudates	5.61 ± 1.20	1.37 ± 0.54	3.53 ± 1.10	1.24 ± 0.71
ETDRS, 1991	[31]	2,709	8	High	Hard exudates	5.44 ± 1.38	1.46 ± 0.48	3.30 ± 1.25	1.28 ± 0.33
SN-DREAMS. 2017	[24]	1,414	8	Moderate	CSME	5.12 ± 0.95	1.45 ± 0.78	3.45 ± 1.31	1.54 ± 0.53
CURES, 2005	[32]	1,736	9	High	Any DR	5.37 ± 1.66	1.39 ± 0.86	3.20 ± 1.16	1.47 ± 0.98
DCCT/EDIC, 2004	[36]	1,441	8	Moderate	DR severity	5.40 ± 1.15	1.34 ± 0.67	3.35 ± 1.45	1.25 ± 0.50
AusDiab, 2003	[29]	2,177	8	High	Any DR	5.50 ± 1.03	1.81 ± 0.36	NA	NA
Javadi et al., 2009	[27]	7,989	8	High	Any DR	5.24 ± 0.86	1.47 ± 0.74	3.32 ± 1.41	1.05 ± 0.21
ARIC, 2016	[33]	1,600	8	High	Hard exudates	5.43 ± 1.18	NA	3.53 ± 1.47	NA
Hoorn, 2003	[37]	626	9	Moderate	Hard exudates	5.57 ± 1.25	1.31 ± 0.51	3.55 ± 1.04	1.23 ± 0.52
	Prospective studies								
WESDR, 2008	[22]	2,366	8	High	Hard exudates	5.61 ± 1.20	1.37 ± 0.54	3.53 ± 1.10	1.24 ± 0.21
DCCT/EDIC, 2004	[36]	1,441	8	Moderate	CSME	5.14 ± 0.75	NA	3.66 ± 1.03	NA
Cohen et al., 1999	[25]	497	8	Moderate	PDR	5.01 ± 1.17	1.21 ± 3.01	3.16 ± 0.96	1.34 ± 0.45

Summary of diverse clinical trials on lipid markers and diabetic retinopathy. The quality assessment of each studies was accessed and scored by using the Newcastle–Ottawa Quality (NOS) Assessment Scale. This scale varied from 0 to 9 stars, which indicated that studies were graded as good if they met greater than 8 criteria. All the following criteria for enrolling the clinical trials had to be met: (1) the design was a case–control study in humans, reporting on two outcome groups: one with DR and one without DR; (2) published observational studies with data on both patients with DR and healthy age-and sex-matched controls; (3) study provided the detailed data in both cases and controls, including the levels of other serum lipids; (4) the healthy controls were clearly described. If study with data published more than once or using the same subjects, we considered all publications for data abstraction, only the article with adequate study strategy was chosen. In addition, studies were excluded if they were reviews, clinical trials, case report, animals’ experiments and discussion papers

*Abbreviation*s: *CSME* Clinically significant macular edema, *TC* total cholesterol, *TG* triglyceride, *LDL-C* low-density lipoprotein cholesterol, *HDL-C* high-density lipoprotein cholesterol, *DR* Diabetic retinopathy, *NPDR* Non-proliferative diabetic retinopathy, *PDR* Proliferative diabetic retinopathy, *NA* Not applicable

## Evidence of lipid-lowering strategies in diabetic retinopathy therapy

Despite the discordant correlation between hyperlipidemia and diabetic retinopathy, belief in a positive correlation leads to the attention shifting towards implementing lipid-lowering strategy in preventing diabetic retinopathy. Unexpectedly, some early clinical investigations, using atromid and clofibrate, failed to improve the visual function in patients, though they obtained a beneficial effect on retinal hard exudate. However, since statin could reduce total cholesterol and LDL-C, while fibrate could decrease TG, the role of these two lipid-lowering agents on modulating serum lipid levels and the progression of diabetic retinopathy has obtained massive attention.

### Statins

The earliest small non-comparative studies, conducted by Gordon et al. in 1991, firstly suggested that the aggressive therapy of diabetic patients with hyperlipidemia might have a beneficial effect on diabetic retinopathy. The study, which contained only six diabetes patients who took pravastatin, showed that all the patients presented improvement in hard exudate and four of them presented improvement in micro-aneurysms [38]. Subsequently, several randomized clinical trials aiming at investigating statin in diabetic retinopathy treatment were published. For instance, one study, enrolling 50 patients with hyperlipidemia and diabetic retinopathy, demonstrated that patients who took simvastatin 20 mg daily had improvement of diabetic retinopathy and less incidence of worsening visual acuity assessed by fluorescein angiography [39]. Likewise, another study, enrolling 30 diabetic patients with hyperlipidemia and hard exudate, also demonstrated that the patients who took atorvastatin presented improvement of hyperlipidemia and reduction in hard exudate. However, the researchers did not observe significant differences in improving CSME regression [40]. Though these trials were clearly limited by small sample of participants, the results strongly suggested the importance of reducing LDL-C and total cholesterol in the subjects with diabetic retinopathy.

In the next several decades, the relationship has been replicated in several large-scale studies from different countries, revealing lipid-lowering therapy is correlated with the improvement of diabetic retinopathy. The Steno-2 Study (S-2-S) is a randomized clinical trial focusing on multiple markers, including glycemic, blood pressure, and serum lipid profiles. During about 13 years of follow-up, 160 diabetic subjects displayed persistent microalbuminuria. Intensive treatment of statin was correlated with an approximately 50% reduction in the development of diabetic macular edema [41]. Recently in 2017, Chung et al. conducted a study enrolling the medical records of 110 diabetic subjects who were randomized to treatment with statin or placebo. After analysis, though statin presented little significant effect on inhibiting the progression of diabetic retinopathy, the macular edema was showed within 30% of subjects with statin and 50% of subjects without statin, indicating that the lipid-lowering treatment with statin inhibits the progression of diabetic macular edema [42]. In last year, a clinical trial containing more than 1,648,300 diabetic individuals demonstrated that under statin therapy, the patients presented a significantly reduced prevalence of diabetic retinopathy, NPDR, PDR, and vitreous hemorrhage, revealing that the statin therapy is necessary and essential for the vision-threatening therapy in participants with hyperlipidemia and hyperglycemia [43]. More recently in 2020, Pranata et al. performed a systematic literature search and demonstrated that statin treatment was strongly correlated with a remarkably decreased risk of diabetic retinopathy. Moreover, statin treatment also lowered the necessary for the therapy with intravitreal injection and vitrectomy [44]. Similar conclusions could also be seen in another study which aimed to assess the function of lipid-lowering agents on diabetic retinopathy and its related complications requiring intervention [45].

Nevertheless, some other studies question the benefit of statin with respect to diabetic retinopathy therapy. For instance, a case–control study, containing 684 subjects, demonstrated no significant correlation between statin therapy and diabetic retinopathy [46]. Similar outcomes could be found in another large-scale clinical trial named Collaborative Atorvastatin Diabetes Study (CADS). The trial, containing 2,838 subjects, is a series trial of lipid-lowering treatment focusing on the primary prevention of cardiovascular diseases under diabetic status. By analysis, the authors found that atorvastatin showed greatest function in lowering the prevalence of first cardiovascular disease events, whereas it had no significant effect on preventing diabetic retinopathy [47]. Taken together, clinical management of hyperlipidemia with statin seems to slow the risk of diabetic retinopathy, and aggressive statin treatment may alter the development of diabetic retinopathy.

### Fibrates

Increasing data has shown the modulatory role of fibrates in patients of diabetic retinopathy, revealing that fibrates embrace the function of inhibiting the progression diabetic retinopathy and its complications. Among multiple studies, the eye-catching results of two large-scale independent clinical surveys were published as the landmark importance of lipid-lowering treatment in patients with diabetic retinopathy. To be more specific, one of the studies is named Actions of Control Cardiovascular Risk in Diabetes (ACCORD) study which is a randomized clinical trial that assesses the management of serum glucose, circulating lipid profiles, and blood pressure in more than 10,250 diabetic subjects with statin and fenofibrate together. After analysis, fenofibrate could significantly prevent the progression of diabetic retinopathy. Combination medication of fibrate and statin further reduced the severity of diabetic retinopathy by approximately 40% compared to those within subjects using simvastatin alone [48, 49]. Similarly, the FIELD study focused on evaluating fenofibrate in cardiovascular disease events in 9,795 diabetic participants. Notably, the authors found a significant reduction in the tertiary outcome of the participants requiring laser photocoagulation, and fenofibrate could decrease the rate of first laser treatment by 31% [50]. Furthermore, in Ophthalmology sub-trial, which contained 1,012 patients without diabetic retinopathy at baseline, the authors demonstrated that the pathogenic development of diabetic retinopathy was significantly modulated by fenofibrate. Additionally, fenofibrate treatment was also correlated with about 20% reduction in the primary endpoint and approximately 60% reduction in the prevalence of macular edema. Over the later five years, patients with fenofibrate were less inclined to laser therapy, revealing that the long-term and relatively extensive dose of fenofibrate could prevent microvascular endpoints [51]. In 2018, Kawasaki et al. also confirmed that among 69,070 subjects with type 2 diabetes, using lipid-lowering agents was associated with lowered risk of diabetic retinopathy prevalence, as well as the decreased incidence of diabetic macular edema [52].

Notably, these two similar results need further discussions. In details, both ACCORD trial and FIELD trial failed to demonstrate that fenofibrate could reduce macrovascular events, but did found a benefit for microvascular events, such as diabetic retinopathy and its complications, indicating that fenofibrate could act on unconventional mechanism to modulate the progression of diabetic retinopathy. Otherwise, in both two trials, the effects on diabetic retinopathy were independent of the regulatory functions of fenofibrates on serum lipid profiles, again suggesting a new protective pathway such as inflammation or endothelial dysfunction. More recently, Shi et al. conducted a meta-analysis, including 8 randomized clinical trials containing 13,454 subjects, and provided evidence for the lipid-lowering agents in treating diabetic retinopathy and its major complications. The results revealed that lipid-lowering agents could significantly reduce the risk of diabetic retinopathy; in addition, these agents might have protective function on diabetic macular oedema [53].

Conclusively, the data provides the evidence for lipid-lowering treatment, fenofibrate in particular, as adjunctive treatment for diabetic retinopathy beyond the traditional strategies of tight management of blood glucose and blood pressure. Given that the previous guideline, named the Australian National Health and Medical Research Council Guidelines for the controlling of diabetic retinopathy, has recommended that physicians should undertake the serum lipid-lowering agents to inhibit the progression of diabetic macular oedema, especially in patients with extensive hard exudate deposition [54], large clinical trials are required to further confirm how to use lipid-lowering agents could bring more benefit for the patients.

### Other potential therapeutic strategies

Owing to technological developments, breakthroughs have been put forward to show the potential therapeutic strategies in improving diabetic retinopathy. First, the role of cholesterol intake from daily diet in exudative maculopathy has been assessed in some studies. For instance, a study containing 8 patients demonstrated that the progression of retinal exudative maculopathy was suppressed after a high-carbohydrate and low-cholesterol diet, indicating that the content of cholesterol plays a vital role in regulating diabetic retinopathy [55]. Consistent with this notion, another factor is the serum level of linoleic acid. An investigation enrolling 137 patients found that patients with retinal exudative maculopathy had a lower percentage of linoleic acid within total cholesterol esters compared to those in the patients without retinal exudative maculopathy [56]. Thereby, the management of daily diet is of great significance in treating diabetic retinopathy.

Second, the effect of nuclear receptors on modulating the progression of diabetic retinopathy has obtained attention. Among multiple receptors, PPAR-γ is considered as one of the most important therapeutic targets in diabetic retinopathy [57]. As shown in previous studies, PPAR-γ could be stimulated by the omega-3 polyunsaturated free acids (PUFAs) and subsequently induced the PUFA-mediated benefit in diabetic retinopathy. The researchers administered omega-3 PUFAs during the neo-vascular stage in mice with oxygen-induced retinopathy and found that the PUFAs induced more than 40% reduced neovascularization [58]. Similar with this conclusion, Damavandi et al. demonstrated that the serum insulin and the LDL-C/HDL-C ratio lowered obviously in patients using PUFAs compared with the control individuals, indicating that consumption of PUFAs could lower circulating insulin and LDL-C/HDL-C ratio in patients with type 2 diabetes mellitus [59]. Recently, a meta-analysis conducted by Sepidarkish et al. showed that co-supplementation with PUFAs and vitamin E increased the levels of nitric oxide and total anti-oxidant capacity, suggesting that PUFAs suppresses the oxidative stress that could be considered as a novel mechanism whereby PUFAs modulates the development of diabetes and diabetic retinopathy [60].

On the other hand, the PPAR-γ agonist, such as rosiglitazone, was verified to significantly delay the progression of proliferative diabetic retinopathy, as the rates of proliferative diabetic retinopathy occurred in 19.2% in patients using rosiglitazone and 47.4% in patients using placebo. Meanwhile, fewer eyes within the subjects using rosiglitazone presented more than three lines of visual acuity loss [61]. Otherwise, some lipid-lowering agents, such as fibrates, act as activators of PPAR-α and resultantly modulate the progression of diabetic retinopathy [62]. This effect may guide us to further understand the beneficial function of fibrate on diabetic retinopathy.

## Relationship between novel serum biomarkers and diabetic retinopathy

In addition to the traditional biomarkers of serum lipid profiles, various novel biomarkers have obtained attention to further explore the mechanism whereby dyslipidemia influences diabetic retinopathy.

### Lipoprotein (a)

Lipoprotein (a) [Lp(a)] is a novel serum lipoprotein which has been verified to be correlated with cardiovascular diseases, such as atherosclerosis, acute coronary syndromes, and other coronary heart diseases [63]. Accumulating clinical trials focusing on the important role of Lp(a) in diabetic retinopathy put forward discordant results. For instance, in several studies containing the type 1 diabetic patients, the authors found a positive relationship between higher circulating levels of Lp(a) with the pathogenic development of diabetes [64, 65], while an equivalent number of trials did not demonstrated any significant association [66, 67]. On the other hand, the results of some studies are contradictory, since both the positive and the negative relationship between circulating Lp(a) and the development of diabetes and its complications were published [36, 68]. Importantly, several potential mechanisms, which accounts for Lp(a) in modulating the development of diabetic retinopathy have been suggested, such as the increased extent of mesangial cell proliferation, the up-regulation of endothelin, the decreased endothelial cell population, and the down-regulation of nitric oxide affecting vascular tone [69, 70].

### Apolipoproteins

The circulating apolipoprotein profiles seemed to be correlated with the development of diabetic retinopathy, which could be considered as novel biomarkers of diabetic retinopathy. In a cross-sectional clinical survey, which contained 224 diabetic participants, the authors detected both traditional lipid biomarkers and non-traditional biomarkers, including ApoA1, ApoA5, ApoC3, and the ratio of ApoA1 to ApoB, and inflammatory cytokines under the fasting status. After analysis, they found that diabetic retinopathy existed in approximately 60% of the diabetic patients. When adjusted for sex, age, blood pressure, duration of diabetes, and individual medications, only serum levels of HDL-C were inversely associated with the severity of diabetic retinopathy; consistently, the serum levels of ApoA1 were confirmed to be inversely correlated with the pathogenic progession of diabetic retinopathy. By contrast, the serum levels of ApoB, ApoC3, and the ratio of ApoA1 to ApoB had positive correlations with diabetic retinopathy. The serum levels of ApoA5 showed no association with the disease [71]. Another trial using a prediction model for diabetic retinopathy which encompassing the traditional risk biomarkers for diabetic retinopathy including blood glucose, blood pressure, and serum lipid profiles. With in-depth investigations, the serum levels of traditional lipid profiles slightly increased, but the apolipoproteins collectively improved the area by approximately 8%. A combination of traditional lipid profiles and apolipoproteins showed improvement by more than 7% [72].

It is worth noting that these reports seem to be biological plausibility. As described, ApoA1 is the major structural and functional protein within HDL particle, which has been verified to exert beneficial effects on cardiovascular diseases and inflammatory diseases due to the antioxidant and anti-inflammatory functions [73]. Supporting this notion, evidence showed that within retinal pigment epithelium, the expression level of ApoA1 was higher in the vitreous body isolated from the diabetic subjects compared to those in patients without diagnosed diabetes, providing evidence of a protective function of ApoA1 against the lipo-toxicity and lipid deposition [74]. By immuno-histochemical research and the immunofluorescence research, the authors observed that the intracellular ApoA1 protein localized on the ganglion cell layer, retinal pigment epithelium, and rod photoreceptor inner, indicating that retina makes use of an intracellular mechanism of lipid profiles transportation [75].

ApoB, the major apolipoprotein within VLDL particle and LDL particle, is correlated with the atherosclerosis and its related coronary diseases [76]. Moreover, ApoB itself has also been confirmed to have the positive relationship with the severity of diabetic retinopathy [77]. By the immunostaining research, the intracellular level of ApoB was increased within the LDL particle during the promotion of diabetic retinopathy, suggesting that ApoB could be used as a novel biomarker of diabetic retinopathy [78].

The circulating levels of diverse apolipoproteins were more predictive for the progression of diabetic retinopathy compared to the serum levels of total cholesterol or LDL-C alone. For example, ApoA1 is associated with the quantity of HDL particle. Thus, the HDL complex, on the other hand, has been verified to be heterogeneous and their function is strongly affected by ApoA1. Otherwise, ApoB is one of the major proteins within the atherogenic lipoproteins in circulation, such as intermediate-density lipoprotein (IDL) and Lp(a). However, given that these apolipoproteins are not detected regularly in clinical practice, it is necessary to conduct more studies to further research the relationship between these biomarkers and diabetic retinopathy. Conclusively, the association between lipid burden with the apolipoprotein could potentially account for the relationship between dyslipidemia with the progression of diabetic retinopathy.

### Some inflammatory cytokines

Some traditional pro-inflammatory factors, such as C-reactive protein (CRP) and IL-1β, is being identified as the novel therapeutic targets of diabetic retinopathy [57, 79]. Accumulating evidence from large clinical trials has shed light on the effect. For instance, the Justification for the Use of Statins in Primary Prevention: an Intervention Trial Evaluating Rosuvastatin (JUPITER) trial found that higher serum levels of CRP and IL-1β were correlated with diabetic retinopathy. Statins exert their therapeutic functions through lowering serum levels of CRP and IL-1β [80]. Furthermore, in another large population-based cohort, named the Hoorn Study, the authors analyzed 625 patients and verified that the higher circulating levels of CRP were associated with the risk and the development of diabetic retinopathy and its complications [37]. Similar relationship could be observed in another independent research, which showed that CRP embraced a positive relationship with diabetic retinopathy [35]. Recently, in another independent longitudinal survey of diabetic patients, the authors identified that IL-1β had a positive relationship with the development of diabetic retinopathy [81]. In a word, these inflammatory biomarkers could be considered as the predictors of diabetic retinopathy, and the anti-inflammatory agents may exert the beneficial functions.

### Fibroblast growth factor 21 (FGF21)

FGF21 is belonged to the FGF19 protein subfamily and is a recently discovered cytokine within circulation. Emerging evidence has demonstrated that the circulating level of FGF21 is increased significantly in multiple cardio-metabolic disorders, embracing an important role in regulating carbohydrate and lipid metabolism, consequently affecting the pathological development of these disorders [82]. Particularly, in the participants with type 2 diabetes, FGF21 has been verified to reduce body weight and ameliorate the hyperlipidemia. By contrast, in mice with both obesity and diabetes, FGF21 reduced serum levels of TG by modulating the lipoprotein catabolism and maintaining the phospholipid homeostasis within adipose tissue [83]. Furthermore, FGF21 also increases lipid utilization in response to amino acid starvation [84].

With in-depth investigation, it has been demonstrated that FGF21 could modulate the activity of proliferator-activated nuclear receptor δ (PPAR-δ), so FGF21 is considered as a crucial PPAR-δ agonist which ameliorates metabolic disorders [85]. On the other hand, FGF21 could also regulate PPAR-α and resultantly affect body fat content. In insulin-deficient mice with diabetes, the authors found that the FGF21 suppressed the secretion of pro-inflammatory cytokines, enhanced retinal antioxidant defense system, restored disorganized cone photoreceptor segments, and improved the retinal function [86]. By deepen research, FGF21 also modulated the synthesis and secretion of adiponectin, which could reversely mediate the function of FGF21 in blood glucose and lipid metabolism [87]. Similarly, Fu et al. confirmed that the adiponectin-mediated FGF21 was correlated with a series of metabolic retinal disorders, inhibiting ocular neovascularization in mice [88]. Taken together, these results strongly suggest that FGF21 could be considered as a novel serum biomarker to predict the development of diabetic retinopathy.

## The unclarified questions and further investigations

Despite increasing results of current extensive study, the accurate relationship between hyperlipidemia and diabetic retinopathy still remains unclarified. Importantly, this question could be taken into account with the oversimplified results of epidemiologic investigations. As reported previously, the serum lipid profiles contain diverse molecules with multiple biological functions, which are dissimilar with other simple risk markers, such as blood pressure and blood glucose. On the other hand, according to the results of epidemiologic surveys, the traditional biomarkers of lipid profiles are not associated with diabetic retinopathy. Consistently, the lipid-lowering therapy has also been put forward to ameliorate diabetic retinopathy which is independent of their modulatory function on serum lipids.

On the other hand, the downstream products of lipid metabolism could also modulate the process of lipid metabolism in turn through specific biochemical mechanisms, indicating that these downstream products could be suitable as the predictors of diseases [89]. Since the therapeutic functions of fibrates on diabetic retinopathy have been demonstrated to be separate of regulating the risk of hypertension, hyperlipidemia, and hyperglycemia, the correlation of diabetic retinopathy with circulating lipid profiles may be beyond the traditional risk markers, and these potential mechanisms need to be further clarified.

Moreover, the lipid-lowering medicines, particularly the statin, ezetimibe, and fibrates, are verified to be effective in improving diabetic retinopathy. However, these results were obtained from sub-analyses of clinical trials which evaluated the cardiovascular end points. Thus, large-scale clinical trials of the therapeutic effect of fibrate or statin as primary prevention for diabetic retinopathy remain lacking and especially urgent. On the other hand, the lack of clinicians who could make the decision in clinical practice, presents the variability in therapies whereby clinicians achieve strict control of blood pressure and blood glycemic. As a consequence, a novel approach is also needed currently. Among all the therapies under investigation and development, the lipid-lowering treatment embraces eye-catching promise which could add to the present clinical therapeutic strategy.

Finally, given that the current lipid-lowering agents are with significant adverse effects, another essential issue is collecting and identifying the profiles of hospitalized patients who would benefit most from the lipid-lowering therapy. In addition to the traditional serum lipid levels which alone are inadequate, the novel serum biomarkers of lipid profiles and inflammatory reactions, or the endothelial dysfunction are potentially related.

## Study strength and limitations

Several limitations in this comprehensive review should be denoted. First, the serum lipid levels were the only outcome assessed, whether treatment with lipid-lowering agents achieved a greater reduction in the risk of diabetic retinopathy are going to require further study. Second, some investigations enrolling relatively small sample size, as less than 500 subjects, which may not embrace the strong evidence enough. Third, the molecular mechanism of lipid-lowering therapy affecting lipid metabolism in patients with diabetic retinopathy has not been reported yet. Thus, the relationship between using lipid-lowering agents, such as statins, fibrates, and ezetimibe, with lipid metabolism in patients with diabetic retinopathy needs further investigation.

Nevertheless, the current review also has its strengths to elucidate the correlation between the classical lipid biomarkers and diabetic retinopathy. Additionally, the lipid-lowering treatments could be identified as one of the most potential therapeutic agents for diabetic retinopathy. By comparison to other single-country or single-race trial, our comprehensive review could increase the generalizability by combining and analyzing the data and conclusions from several different studies across different continents. Thereby, our review could provide a relatively precise assessment on the relationship between dyslipidemia and diabetic retinopathy. Moreover, the favorable effects of lipid-lowering agents on modulating the risk of diabetic retinopathy by enhancing the statistical significances and resolutions.

## Conclusions and future perspectives

The present review sheds light on that even if the lack of accurate correlation between the classical lipid biomarkers and diabetic retinopathy, the lipid-lowering treatments could be identified as one of the most potential therapeutic agents for diabetic retinopathy. The lipid-lowering agents, including statin and fenofibrate, function through various pathways which might be attached to the promotion of atherosclerosis and its related coronary heart diseases, suggesting novel mechanisms by which the agents prevent diabetic retinopathy, such as promoting systemic inflammatory reactions. Future investigations to illustrate these mechanisms embrace promise for the promotion of novel types of agents. As a consequence, the inclusion of serum lipid management could be considered as another important arm in treating diabetic retinopathy.

## Data Availability

Not applicable.

## Abbreviations

LDL-C: Low-density lipoprotein cholesterol
VLDL-C: Very low-density lipoprotein cholesterol
TG: Triglyceride
HDL-C: High-density lipoprotein cholesterol
Lp(a): Lipoprotein (a)
ApoA1: Apolipoprotein A1
ApoA5: Apolipoprotein A5
ApoB: Apolipoprotein B
ApoC3: Apolipoprotein C3
RCT: Reverse cholesterol transport
NPDR: Non-proliferative diabetic retinopathy
PDR: Proliferative diabetic retinopathy
FGF: Fibroblast growth factor
MACE: Major adverse cardiovascular event
WESDR: The Wisconsin Epidemiologic Study of Diabetic Retinopathy
SRT: The Sorbinil Retinopathy Trial
AusDiab Study: The Australian Diabetes, Obesity and Lifestyle Study
SMES: Singapore Malay Eye Study
BMI: Body mass index
ETDRS: Early Treatment of Diabetic Retinopathy Study
CURES: Chennai Urban Rural Study
SN-DREAMS: Sankara Nethralaya Diabetic Retinopathy Epidemiology and Molecular Genetic Study
S-2-S: Steno-2 Study
CADS: Collaborative Atorvastatin Diabetes Study
ACCORD: Actions of Control Cardiovascular Risk in Diabetes
PUFAs: The omega-3 polyunsaturated free acids
IDL: Intermediate-density lipoprotein
JUPITER: The Use of Statins in Primary Prevention: an Intervention Trial Evaluating Rosuvastatin trial
PPAR: Proliferator-activated nuclear receptor
